# Dosage and cycle effects of dacarbazine (DTIC) and fotemustine on O6-alkylguanine-DNA alkyltransferase in human peripheral blood mononuclear cells.

**DOI:** 10.1038/bjc.1993.42

**Published:** 1993-02

**Authors:** S. M. Lee, N. Thatcher, M. Dougal, G. P. Margison

**Affiliations:** CRC Dept of Carcinogenesis, Paterson Institute for Cancer Research, Manchester, UK.

## Abstract

There is increasing experimental evidence to suggest that endogenous expression of O6-alkylguanine-DNA-alkyltransferase (ATase) is a major factor in cellular resistance to certain chemotherapeutic agents including dacarbazine (DTIC). We have recently shown wide interindividual variation in the depletion and subsequent regeneration of ATase in peripheral blood mononuclear cells (PMCs) following DTIC and this has now been extended to ascertain whether or not depletion is related to dosage of DTIC used and repeated treatment cycles of chemotherapy. ATase levels were measured in three groups of 25 patients (pts) up to 24 h after receiving DTIC at 400 mg m-2, 500 mg m-2 or 800 mg m-2. Each group also received fotemustine (100 mg m-2), 4 h after DTIC. The lowest extent of ATase depletion (highest nadir ATase) was seen in patients receiving 400 mg m-2. The mean nadir ATase, expressed as a percentage of pre-treatment ATase, was respectively 56.3%, 26.4% and 23.9% for 400 mg m-2, 500 mg m-2 and 800 mg m-2. The median nadir of ATase activity for pts receiving 800 mg m-2 pts was at 4-6 h and for pts given lower doses it was at 2-3 h. In addition, repeated measures analysis of variance of observations before chemotherapy, then at 2, 3, 4, 6 and 18 h after chemotherapy provides some evidence that ATase was depleted to a lesser extent after cycle 1 than after subsequent cycles (P = 0.025). It also provides evidence that the change in ATase activity over time varied with dose and cycle. The findings can be interpreted on the basis of a dosage-dependent metabolism of DTIC to an agent capable of methylation of DNA and subsequent depletion of PMC ATase: with higher DTIC doses, the extent of ATase depletion may be limited by the pharmacokinetics of DTIC metabolism. PMC ATase was measured in another group of 8 pts at various times after receiving only fotemustine (100 mg m-2) and in contrast to DTIC, no ATase depletion was seen suggesting that insufficient concentrations of fotemustine and/or its metabolites were available to react with DNA to produce a depletion of PMC ATase activity.


Br. J. Cancer (1993), 67, 216-221 © Macmillan Press Ltd., 1993

### Dosage and cycle effects of dacarbazine (DTIC) and fotemustine on $O^6$-alkylguanine-DNA alkyltransferase in human peripheral blood mononuclear cells

S.M.Lee[1,2], N. Thatcher[2], M. Dougal[3] & G.P. Margison[1]

[1]*CRC Dept of Carcinogenesis, Paterson Institute for Cancer Research,* [2]*CRC Department of Medical Oncology, and* [3]*Department of Medical Statistics, Christie Hospital NHS Trust, Manchester M20 9BX, UK.*

**Summary** There is increasing experimental evidence to suggest that endogenous expression of $O^6$-alkylguanine-DNA-alkyltransferase (ATase) is a major factor in cellular resistance to certain chemotherapeutic agents including dacarbazine (DTIC). We have recently shown wide interindividual variation in the depletion and subsequent regeneration of ATase in peripheral blood mononuclear cells (PMCs) following DTIC and this has now been extended to ascertain whether or not depletion is related to dosage of DTIC used and repeated treatment cycles of chemotherapy. ATase levels were measured in three groups of 25 patients (pts) up to 24 h after receiving DTIC at 400 mg m$^{-2}$, 500 mg m$^{-2}$ or 800 mg m$^{-2}$. Each group also received fotemustine (100 mg m$^{-2}$), 4 h after DTIC. The lowest extent of ATase depletion (highest nadir ATase) was seen in patients receiving 400 mg m$^{-2}$. The mean nadir ATase, expressed as a percentage of pre-treatment ATase, was respectively 56.3%, 26.4% and 23.9% for 400 mg m$^{-2}$, 500 mg m$^{-2}$ and 800 mg m$^{-2}$. The median nadir of ATase activity for pts receiving 800 mg m$^{-2}$ pts was at 4–6 h and for pts given lower doses it was at 2–3 h. In addition, repeated measures analysis of variance of observations before chemotherapy, then at 2, 3, 4, 6 and 18 h after chemotherapy provides some evidence that ATase was depleted to a lesser extent after cycle 1 than after subsequent cycles ($P = 0.025$). It also provides evidence that the change in ATase activity over time varied with dose and cycle. The findings can be interpreted on the basis of a dosage-dependent metabolism of DTIC to an agent capable of methylation of DNA and subsequent depletion of PMC ATase: with higher DTIC doses, the extent of ATase depletion may be limited by the pharmacokinetics of DTIC metabolism. PMC ATase was measured in another group of 8 pts at various times after receiving only fotemustine (100 mg m$^{-2}$) and in contrast to DTIC, no ATase depletion was seen suggesting that insufficient concentrations of fotemustine and/or its metabolites were available to react with DNA to produce a depletion of PMC ATase activity.

In the treatment of metastatic melanoma, dacarbazine [5-(3,3-dimethyl-1-triazeno)imidazole-4-carboxamide; DTIC] is considered the single most effective chemotherapeutic agent available (Balch *et al.*, 1989; Comis, 1976). It undergoes metabolic N-demethylation to give the cytotoxic monomethyl triazene, 5-(3-methyl-1-triazeno)imidazole-4-carboxamide (MTIC) which methylates DNA, producing among 12 other lesions, $O^6$-methylguanine (Meer *et al.*, 1986). There is increasing evidence to suggest that $O^6$-methylguanine is the principal cytotoxic event following DTIC and that $O^6$-alkylguanine-DNA alkyltransferase (ATase) gene expression may be a major factor in cellular resistance to such agents. ATase is able to transfer the methyl group from the $O^6$ position of guanine to an internal cysteine residue in an auto-inactivating stoichiometric reaction. Experimental models using ATase-deficient cell lines or xenografts show them to be more sensitive to DTIC than lines or xenografts with high activity (Catapano *et al.*, 1987; D'Incalci *et al.*, 1988; Foster *et al.*, 1990; Gibson *et al.*, 1986*a*; Hayward and Parsons, 1984; Lunn & Harris, 1988). The strongest evidence for the cytotoxic effects of $O^6$-alkylguanine in DNA comes from ATase cDNA transfection experiments which show that expression of prokaryotic or eukaryotic ATase cDNA in mammalian cells protects them against the toxic effects to these agents (Brennand and Margison, 1986; Jelinek *et al.*, 1988; Kataoka *et al.*, 1986; Samson *et al.*, 1986; Kaina *et al.*, 1991).

We recently showed that there was wide interindividual variation in the DTIC-mediated depletion and subsequent recovery of ATase levels in human peripheral blood cells (Lee *et al.*, 1991*a*) and this work has now been extended to explore whether or not the rate and extent of depletion and regeneration of ATase activity is related to the dosage of DTIC used or the number of treatment cycles. Identifying the time to reach the ATase nadir and the extent of ATase depletion with different DTIC dosage may have important therapeutic implications especially when DTIC is combined with the subsequent administration of a chloroethylating nitrosourea. In this case, drug resistance appears to involve the same ATase DNA repair enzymes which remove the chloroethyl lesions from the $O^6$-position of guanine, thereby preventing the formation of a cytotoxic DNA interstrand cross-link (see Lee *et al.*, 1991*a*). Theoretically, enhanced therapeutic effects might be obtained when the nitrosourea is administered at the nadir of ATase activity following DTIC treatment assuming that the effect in peripheral mononuclear cells reflects that of tumour tissues.

Correspondence: S.M. Lee.
Received 20 March and in revised form 9 September 1992.

### Materials and methods

### *Patients and blood samples*

Blood samples were collected from 30 treatment cycles of 25 pts with metastatic melanoma treated with sequential DTIC and fotemustine chemotherapy. Approval was obtained from the local ethical committee and all pts gave informed consent for the study. Pts received DTIC at fixed doses (for each pt) of 400, 500 or 800 mg m$^{-2}$ by i.v. infusion followed by fotemustine 100 mg m$^{-2}$, 4 h later. Treatment was repeated every 28 days and the number of cycles given depended on the individual pts response. Blood samples were collected just before chemotherapy and at 1, 2, 3, 4, 6 and 18 h after DTIC infusion; in addition, 5 h samples were collected for the 400 mg m$^{-2}$ patients. Blood was drawn into a 20 ml univeral container containing 0.5% EDTA and stored at 4°C before isolation of PMCs. Fourteen sets of samples from were also collected from another group of 8 pts with metastatic melanoma receiving only fotemustine (100 mg m$^{-2}$).

When the study was initiated, no effect of treatment cycle on ATase concentrations was anticipated. As a result samples were not taken from pts on the same cycles. Some pts had samples taken on cycle 1, other on cycle 2, etc. and some pts had samples taken on more than one cycle. In the event, the cycle in which ATase was measured had an effect on the ATase level and as a result the dose and cycle effects are confounded. To avoid this, samples were randomly selected for each analysis so that no more than one set of observations was used from a patient. As this resulted in loss of data, we present this aspect of the study as a guide to possible future studies. The principal method of statistical analysis was analysis of variance, with repeated measures analysis of variance used for analysing the changes over time and 'survival' analysis for estimating the time to the ATase nadir. The computer software package BMDP was used for most analyses.

### *Isolation of mononuclear cells, ATase extraction and assay*

This was carried out as described previously (Lee *et al.*, 1991*a*). Briefly, the PMCs were isolated by centrifugation on Ficoll (Pharmacia, Uppsala, Sweden) (Boyum, 1968), sonicated and the supernatants were assayed using [$^3$H]-methylated DNA containing 0.01 picomoles O$^6$-methylguanine per μg DNA. Activity was expressed as fmoles methyl transferred to protein per mg of protein. Measurements were in triplicate. The mean ATase values are presented ± standard error of the mean.

### Results

### *PMC ATase activity following DTIC and fotemustine*

The change in ATase activity over time was analysed using repeated measures analysis of variance, unweighted means method (Winer *et al.*, 1991*a*). The factors used were the three doses and the cycles, grouped as cycle 1 and cycles 2 to 6. The level of significance was $P \leqslant 0.05$.

### *Dose effects*

In the three groups of pts receiving 400, 500 and 800 mg m$^{-2}$ DTIC, the mean pretreatment PMC ATase levels and their standard errors before the start of the 1st cycle were 230 ± 16, 233 ± 44 and 211 ± 18 fm mg$^{-1}$ respectively. A one-way analysis of variance revealed no statistically significant difference ($P = 0.936$) and therefore subsequent effects of ATase levels were not influenced by inadvertant preselection bias in the dosage groups. The analysis used all cases with measurements on cycle 1.

Following intravenous DTIC administration, progressive depletion of ATase activity was seen (Figure 1). The changes with time were, as expected from earlier studies (Lee *et al.*,, 1991*a*) very highly significant, but more interestingly so was the interaction between time and dose ($P < 0.0001$). This shows that ATase activity changes differently over time for different doses. The least extensive ATase depletion was generally seen in pts receiving 400 mg m$^{-2}$ (Figure 2). The mean nadir ATase, expressed as a percentage of pretreatment ATase, was 56.3%, 26.4% and 23.9% for 400, 500 and 800 mg m$^{-2}$ respectively (Figure 2) and a two way analysis of variance, which included the effect of the two cycle groups, confirmed that the observed difference in the nadir between the doses of DTIC was significant at the 5% level ($P < 0.005$).

The time to the nadir of ATase activity was analysed like a survival analysis and tested with the log rank test. Cases whose lowest ATase levels were after 6 h can be considered as 'survivors' whether or not the 18 h figure is higher: it is not possible to indicate whether or when in the 12 h interval the nadir has occurred and 6 h is therefore a censored time. Pts who received 400 mg m$^{-2}$ were pooled with pts who received 500 mg m$^{-2}$ so that there were enough cases in each

**Figure 1** Lymphocyte ATase activity before and after chemotherapy with 400 (o), 500 (x) or 800 mg m$^{-2}$ ( + ) DTIC. Fotemustine was administered 4 h after DTIC. Values shown are the means ± standard error. An average of >9, 4 and 10 pts were analysed for each time point in the 400, 500 and 800 mg m$^{-2}$ groups, respectively.

**Figure 2** Relationship between DTIC dosage and nadir ATase activity expressed as % of the pretreatment level. The mean values are shown as a horizontal bar.

group for an effective analysis and seven observations were deleted so that no patient appeared more than once in the analysis. The time to nadir was significantly less for pts receiving 400 mg $m^{-2}$ or 500 mg $m^{-2}$ (median 2–3 h) than 800 mg $m^{-2}$ (median 5–6 h) ($P = 0.0407$).

In less than half of the patients, there was a post nadir increase in ATase levels by 18 h after treatment. In most cases this was slight, ranging from 6% to 30% of the pretreatment levels. However, in two pts receiving 400 mg $m^{-2}$ and one receiving 500 mg $m^{-2}$, recovery was very extensive and rapid, attaining close to pretreatment levels: in the former pts this was associated with an earlier ATase nadir. In order to establish whether the increase in ATase by 18 h was statistically significant, the mean ATase at 18 h was compared with the of the nadir. The nadir was taken as being between 4 and 6 h at which time the means were 99.6 fm/mg and 99.4 fm $mg^{-1}$ respectively: the 18 h mean was 111.7 fm $mg^{-1}$. The difference (12.2 fm $mg^{-1}$) was not significant at the 5% level using the Tukey method for comparing means (Winer *et al.*, 1991*b*). Thus by 18 h the mean ATase had not recovered and in fact in some pts the 18 h value is the lowest recorded, although this does not necessarily indicate that it is the nadir.

### *Cycle effects*

The mean pre-treatment ATase activity would seen to be higher in cycle 1 (220 ± 25 fm $mg^{-1}$) than in cycles 2–6 (171 ± 31 gm $mg^{-1}$) (Figure 3). However, the *P*-value from an analysis of variance is greater than 0.1. Hence the observed difference could be due to chance. Seven observations were deleted so that no patient appeared more than once in the analysis and the listed means were derived from the remaining cases.

**Figure 3** Lymphocyte ATase activity before and after chemotherapy with different treatment cycles of DTIC. Values shown are the means ± 95% confidence interval. There were 15 and 8 pts in the cycle 1( + ) and subsequent combined cycle (x) groups, respectively.

The overall difference in ATase levels between the two cycle groups was significant ($P<0.025$) but the magnitude of the difference greatly depended on the number of hours after CT (group versus time interaction $P<0.0001$). This shows that the effect of cycle changes over the sequence of measurements. The mean nadir ATase levels were 41.7% of the pretreatment level for cycle 1 and 29.8% for cycles 2 to 6 but the previously mentioned 2-way analysis of variance provided inadequate evidence for an effect of cycle at the nadir ($P>0.1$). The analysis and the percentages listed above were based on 23 cases. There was no significant difference between the cycles in the time to nadir using the log rank test ($P=0.285$).

*PMC ATase activity following fotemustine alone*

The mean pre-treatment ATase level for pts receiving fotemustine only was $242 \pm 30$ fm mg$^{-1}$ for cycle 1 and $176 \pm 21$ fm mg$^{-1}$ for cycles 2 and 3 combined. All pts were given three cycles of treatment and most pts were studied on more than one cycle, so the pts which were used in the analysis could act as their own controls. Each patient analysed contributed one observation only to both cycle 1 and the combined cycles 2 and 3 and the means quoted above were for the cases used in the analysis. A Wilcoxon paired sample test was used to test the difference between the cycles and a *P* value of 0.0625 provided inadequate evidence that the observed difference was due to anything other than chance.

The ATase levels at 3–4 h or at 16–18 h after fotemustine were not significantly different from the pretreatment values ($P>0.9$) and there was no significant difference ($P>0.9$) between ATase levels when different treatment cycles were compared (Figure 4).

### Discussion

In the present study, we were able to demonstrate DTIC-induced depletion of ATase activity in human PMCs. This is consistent with the metabolism of DTIC to the monomethyl metabolite, MTIC which is produced in sufficient amounts to react with DNA in PMCs to generate $O^6$-methylguanine. This is stoichiometrically repaired by ATase causing an apparent decrease of PMC ATase activity. The nadir of ATase activity generally occurred later in pts receiving 800 mg m$^{-2}$ than in the lower dosage groups; pts receiving 400 mg m$^{-2}$ seemed to have the lowest extent of ATase depletion (highest ATase nadir) with a mean ATase nadir of 56.3% of pretreatment level. In contrast, pts receiving 500 and 800 mg m$^{-2}$ DTIC had a lower mean nadir PMC activity of 26.4% and 23.9%. This suggests that the pharmacokinetics of DTIC is dose-dependent. Indeed, it has been shown that high-dose DTIC (850–1980 mg m$^{-2}$) follows nonlinear pharmacokinetics with saturation occurring in the metabolism and also a slower distribution and disposition rate when compared to lower dose DTIC (Breithaupt *et al.*, 1982; Buesa & Urrechaga, 1991; Loo *et al.*, 1968; Skibba *et al.*, 1969). The later nadir in ATase activity seen with 800 mg m$^{-2}$ DTIC in contrast to 400 mg m$^{-2}$ may be related to the more protracted production of alkylating metabolites.

Whilst ATase recovery by 18 h was generally not substantial and evident in less than half of the pts, in two pts given 400 mg m$^{-2}$, the ATase nadir was around 2 h and activity recovered rapidly to attain close to pretreatment levels. These results further suggest interindividual differences in the continued availability of methylating metabolites and/or in the *de novo* synthesis rates for ATase. In view of the possible saturable pharmacokinetics with high dose DTIC, it would be clearly interesting to administer DTIC by continuous infusion or pulsed low doses in order to assess whether or not a complete loss of ATase activity could be achieved using PMCs as a target.

Despite wide interindividual variations in pretreatment levels, post-treatment DTIC-induced PMC ATase depletion and subsequent recovery, the data suggested that ATase depletion occurred to a lesser mean extent in treatment cycle 1 compared to subsequent treatment cycles. A similar finding was reported in some pts treated with procarbazine (Sagher *et al.*, 1989). This effect might be a consequence of the initial doses of methylating agent (or in the present case, fotemustine) increasing the capacity for metabolic activation of subsequent doses leading to increased levels of DNA methylation. If a similar increase occurred in tumour cells it might be expected that later treatment cycles might be more therapeutically effective than the first cycle.

Whilst the mean pretreatment ATase activity was apparently reduced in subsequent treatment cycles in comparison to cycle 1, in the present study this was not statistically significant and is unlikely to contribute to the differential extent of ATase depletion in later cycles. If, however, a reduction in the mean pre-treatment ATase levels is observed in a larger group of pts, it might presumably be the result of a drug-mediated selection of PMCs expressing lower levels of ATase, although how this occurs is not clear at present.

No statistically significant change in PMC ATase activity occurred in pts treated with 100 mg m$^{-2}$ fotemustine alone although the possibility that synergistic effect on ATase depletion might have occurred in patients given DTIC prior to fotemustine cannot be excluded. This suggests that

**Figure 4** Mean lymphocyte ATase activity before and after chemotherapy with fotemustine alone (100 mg m$^{-2}$). Values shown are the mean ± standard error of the mean for treatment cycles 1 to 3.

insufficient concentrations of fotemustine or its metabolites were available to react with PMC DNA to produce a detectable lowering of ATase activity. A similar finding was reported for human PMCs treated with low dose BCNU (40–200 mg m$^{-2}$) although a statistically significant reduction in PMC ATase activity was seen after high dose BCNU (350 mg m$^{-2}$) (Gerson, 1989). Experimental models have repeatedly shown that depletion of ATase (approximately 60–90% depletion) can sensitise tumour cells to chloroethylnitrosoureas, resulting in a 2- to 12- fold reduction in the 50% lethal dose of these compounds. Greater extents of sensitisation were seen in tumour cells expressing high ATase activity than cells with low levels of activity (Dolan *et al.*, 1985; Dolan *et al.*, 1991; Gerson *et al.*, 1985; Gibson *et al.*, 1986*b*; Zlotogorski & Erickson, 1984) If ATase is the principal mechanism of tumour cell resistance to methylating and chloroethylating agents and if the results obtained with PMC can be extrapolated to tumour cells, our findings would advocate the use of sequential DTIC then fotemustine treatment (as here) rather then a schedule where DTIC and fotemustine are administered concurrently or in which fotemustine is given before DTIC.

Whilst such extrapolations should be considered with appropriate caution, they should also be assessed in relation to the available relevant clinical data. Thus in melanoma pts, DTIC alone (including high dosage) regularly produces a response rate of 20% (Balch *et al.*, 1989; Comis, 1976; Cowan & Bergsagel, 1971; Pritchard *et al.*, 1980); fotemustine alone produces a 24% response rate (Jacquillat *et al.*, 1990). However in pts treated with sequential DTIC and fotemustine, we achieved an overall response rate of 34% and there was a trend towards higher response rate with pts treated with 800 mg m$^{-2}$ DTIC, followed by 500 mg m$^{-2}$ and 400 mg m$^{-2}$ DTIC respectively (Aamdal *et al.*, 1991; Lee *et al.*, 1991*b*). These results provide circumstantial evidence in support of the use of PMC ATase levels as a monitor for those in tumour tissues. Further support is provided by the extent of the toxic side effects of the treatment. Thus there was a statistically significant dosage-dependent development of severe haematological toxicity ($P < 0.01$) in the three groups of pts analysed (Lee & Thatcher, unpublished data). It is tempting to attribute this to greater DNA alkylation with higher dosage DTIC: bone marrow has one of the lower ATase levels of the human tissues examined so far (Gerson *et al.*, 1986) and this, together with the possibility that ATase depletion might increase with treatment cycle, may account for its greater sensitivity to the toxic effects of DTIC. Indeed we have previously shown that ATase-deficient chloroethylnitrosourea-sensitive murine haemopoietic stem cells transfected with a bacterial ATase gene become highly resistant to the toxic effects of methylating and chloroethylating agents, strongly suggesting that ATase would protect against the haematological effects of these agents (Jelinek *et al.*, 1988); other work (Dumenco *et al.*, 1989) supports this finding.

As the bone marrow is generally the principal target organ for the toxic side effects of these agents, it may be possible to protect this tissue by transfection of human pluripotent stem cells with an ATase cDNA. These cells may be returned to the pts in the course of bone marrow transplantation. If this achieves high levels of ATase expression, one can envisage treating pts with high dose alkylating agents which, assuming a linear dose response curve, might result in the elimination of the tumour but spare the ATase transfected bone marrow precursors.

We thank The Institut de Recherches Internationales Servier for providing fotemustine. This work was supported by the Cancer Research Campaign, United Kingdom.

### References

AAMDAL, S., LEE, S.M., RADFORD, J.A., THATCHER, N., CALABRESI, F., ISRAELS, S.P., KERGER, J., STAMATAKIS, L., KLEEBERG, U.R., BROCKER, E. & GERARD, B. (1991). Sequential administration of dacarbazine (DTIC) and fotemustine in disseminated melanoma. *Proc. Am. Assoc. Cancer Res.*, **32**, 191.

BALCH, C.M., HOUGHTON, A. & PETERS, L. (1989). Cutaneous melanoma. In *Cancer: Principles and Practice of Oncology*, DeVita, V.T., Hellman, S. & Rosenberg, S.A. (eds) pp. 1499–1542. Lippincott: Philadelphia.

BOYUM. (1968). Isolation of mononuclear cells and granulocytes from human blood. *Scand. J. Clin. Lab, Invest.*, **21**, 77–89.

BREITHAUPT, H., DAMMANN, A. & AIGNER, K. (1982). Pharmacokinetics of dacarbazine (DTIC) and its metabolite 5-aminoimidazole-4-carboxamide (AIC) following different dose schedules. *Cancer Chemother. Pharmacol.*, **9**, 103–109.

BRENNAND, J. & MARGISON, G.P. (1986). Reduction of the toxicity and mutagenicity of alkylating agents in mammalian cells harboring the *Escherichia coli* alkyltransferase gene. *Proc. Natl Acad. Sci. USA*, **83**, 6292–6296.

BUESA, J.M. & URRECHAGA, E. (1991). Clinical pharmacokinetics of high-dose DTIC. *Cancer Chemother. Pharmacol.*, **28**, 475–479.

CATAPANO, C.V., BROGGINI, M., ERBA, E., PONTI, M., MARIANI, L., CITTI, L. & D'INCALCI, M. (1987). *In vitro* and *in vivo* methazolostone-induced DNA damage and repair in L1210 leukemia sensitive and resistant to chlorethylnitrosoureas. *Cancer Res.*, **47**, 4884–4889.

COMIS, R.L. (1976). DTIC (NSC-45388) in malignant melanoma: a perspective. *Cancer Treat. Rep.*, **60**, 165–176.

COWAN, D.H. & BERGSAGEL, D.E. (1971). Intermittent treatment of metastatic malignant melanoma with high dose 5-(3,3-dimethyl-1-triazeno)imidazole-4-carboxamide (NSC-45388). *Cancer Chemother. Rep.*, **55**, 175–181.

D'INCALCI, M., CITTI, L., TAVERNA, P. & CATAPANO, C.V. (1988). Importance of DNA repair enzyme O$^6$-alkyltransferase (AT) in cancer chemotherapy. *Cancer Treat. Rev.*, **15**, 279–292.

DOLAN, M.E., CORSICO, C.D. & PEGG, A.E. (1985). Exposure of HeLa cells to O$^6$-alkylguanines increases sensitivity to the cytotoxic effects of alkylating agents. *Biochem. Biophys. Res. Commun.*, **132**, 178–185.

DOLAN, M.E., MITCHELL, R.B., MUMMERT, C., MOSCHEL, R.C. & PEGG, A.E. (1991). Effect of O$^6$-benzylguanine analogues on sensitivity of human tumor cells to the cytotoxic effects of alkylating agents. *Cancer Res.*, **51**, 3367–3372.

DUMENCO, L.L., WARMAN, B., HATZOGLOU, M., LIM, I.K., ABBOUD, S.L. & GERSON, S. (1989). Increase in nitrosourea resistance in mammalian cells by retrovirally mediated gene transfer of bacterial O$^6$-alkylguanine-DNA alkyltransferase. *Cancer Res.*, **49**, 6044–6051.

FOSTER, B.J., NEWELL, D.R., LUNN, J.M., JONES, M. & CALVERT, A.H. (1990). Correlation of dacarbazine and CB10-277 activity against human melanoma xenografts with O$^6$-alkyltransferase. *Proc. Am. Assoc. Cancer Res.*, **31**, 401.

GERSON, S.L., TREY, J.E., MILLER, K. & BERGER, N.A. (1986). Comparison of O$^6$-alkylguanine-DNA alkyltansferase activity based on cellular DNA content in human, rat and mouse tissues. *Carcinogenesis*, **7**, 745–749.

GERSON, S.L., TREY, J.E. & MILLER, K. (1988). Potentiation of nitrosourea cytotoxicity in human leukemic cells by inactivation of O$^6$-alkylguanine-DNA alkyltransferase. *Cancer Res.*, **48**, 1521–1527.

GERSON, S.L. (1989). Modulation of human lymphocyte O$^6$-alkylguanine DNA alkyltransferase by streptozotocin *in vivo*, *Cancer Res.*, **49**, 3134–3138.

GIBSON, N.W., HARTLEY, J.A., LAFRANCE, R.J. & VAUGHAN, K. (1986*a*). Differential cytotoxicity and DNA-damaging effects produced in human cells of the Mer$^+$ and Mer$^-$ phenotypes by a series of alkyltriazenylimidazoles. *Carcinogenesis*, **7**, 259–265.

GIBSON, N.W., HARTLEY, J.A., BARNES, D. & ERICKSON, L.C. (1986*b*). Combined effect of streptozotocin and mitozolomide against four human cell lines of the Mer$^+$ phenotype. *Cancer Res.*, **46**, 4995–4998.

HAYWARD, I.P. & PARSONS, P.G. (1984). Comparison of virus reactivation, DNA base damage, and cell cycle effects in autologous melanoma cells resistant to methylating agents. *Cancer Res.*, **44**, 55–58.

JACQUILLAT, C., KHAYAT, D., BANZET, P., WEIL, M., FUMOLEAU, P., AVRIL, M.F., NAMER, M., BONNETERRE, J., KERBRAT, P., BONERANDI, J.J., BUGAT, R., MONTCUQUET, P., CUPISSOL, D. LAUVIN, R., VILMER, C., PRACHE, C. & BIZZARI, J.P. (1990). Final report of the French multicenter phase II study of the nitrosourea fotemustine in 153 evaluable patients with disseminated malignant melanoma including patients with cerebral metastases. *Cancer*, **66**, 1873–1878.

JELINEK, J., KLEIBL, K., DEXTER, T.M. & MARGISON, G.P. (1988). Transfection of murine multi-potent haemopoietic stem cells with an *E.coli.* DNA alkyltransferase gene confers resistance to the toxic effects of alkylating agents. *Carcinogenesis*, **9**, 81–87.

KAINA, B., FRITZ, G., MITRA, S. & COQUERELLE, T. (1991). Transfection and expression of human $O^6$-methylguanine-DNA methyltransferase (MGMT) cDNA in Chinese hamster cells: the role of MGMT in protection against the genotoxic effects of alkylating agents. *Carcinogenesis*, **12**, 1857–1867.

KATAOKA, H., HALL, J. & KARRAN, P. (1986). Complementation of sensitivity to alkylating agents in *Escherichia coli* and Chinese Hamster cells by expression of a cloned bacterial repair gene. *EMBO. J.*, **5**, 3195–3200.

LEE, S.M., THATCHER, N. & MARGISON, G.P. (1991*a*). $O^6$-alkylguanine-DNA alkyltransferase depletion and regeneration in human peripheral lymphocytes following dacarbazine and fotemustine. *Cancer Res.*, **51**, 619–623.

LEE, S.M., SHELBOURN, S.L. & THATCHER, N. (1991*b*). Sequential DTIC and fotemustine in the treatment of metastatic melanoma. *Eur. J. Cancer*, **27**/Supp 2, S160.

LOO, T.L., LUCE, J.K., JARDINE, J.H. & FREI, E, III. (1968). Pharmacologic studies of the antitumour agent 5-(dimethyltriazeno)-imidazole-4-carboxamide. *Cancer Res.*, **28**, 2448–2453.

LUNN, J.M. & HARRIS, A.L. (1988). Cytotoxicity of 5-(3-methyl-1-triazeno)imidazole-4-carboxamide (MTIC) on $Mer^+$, $Mer^+$, $Rem^-$, and $Mer^-$ cell lines: differential potentiation by 3-acetamidobenzamide. *Br. J. Cancer*, **57**, 54–58.

MEER, L., JANZER, R.C., KLEIHUES, P. & KOLAR, G.F. (1986). *In vivo* metabolism and reaction with DNA of the cytostatic agent, 5-(3,3-dimethyl-1-triazeno)imidazole-4-carboxamide (DTIC). *Biochem. Pharmac.*, **35**, 3243–3247.

PRITCHARD, K.I., QUIRT, I.C., COWAN, D.H., OSOBA, D. & KUTAS, G.J. (1980). DTIC therapy in metastatic malignant melanoma: A simplified dose schedule. *Cancer Treat. Rep.*, **64**, 1123–1126.

SAGHER, D., KARRISON, T., SCHWARTZ, J.L., LARSON, R.A. & STRAUSS, B. (1989). Heterogeneity of $O^6$-alkylguanine-DNA alkyltransferase activity in peripheral blood lymphocytes: relationship between this activity in lymphocytes and in lymphoblastoid lines from normal controls and from patients with Hodgkin's disease or non-Hodgkin's lymphoma. *Cancer Res.*, **49**, 5339–5344.

SAMSON, L., DERFLER, B. & WALDSTEIN, E.A. (1986). Supression of human alkylation-repair defects by *Escherichia coli* DNA-repair gene. *Proc. Natl Acad. Sci. USA*, **83**, 5607–5610.

SKIBBA, J.L., RAMIREZ, G., BEAL, D.D. & BRYAN, G.T. (1969). Preliminary clinical trial and the physiologic disposition of 4(5)-(3,3-dimethyl-1-triazeno)imidazole-5(4) carboxamide in man. *Cancer Res.*, **29**, 1944–1951.

WINER, B.J., BROWN, D.R. & MICHELS, K.M. (1991*a*). *Statistical Principles in Experimental Design*. McGraw-Hill: New York pp 497–582.

WINER, B.J., BROWN, D.R. & MICHELS, K.M. (1991*b*). *Statistical Principles in Experimental Design*. McGraw-Hill: New York pp 172–182.

ZLOTOGORSKI, C. & ERICKSON, L.C. (1984). Pretreatment of human colon tumour cells with DNA methylating agents inhibits their ability to repair chloroethyl monoadducts. *Carcinogenesis*, **5**, 83–87.


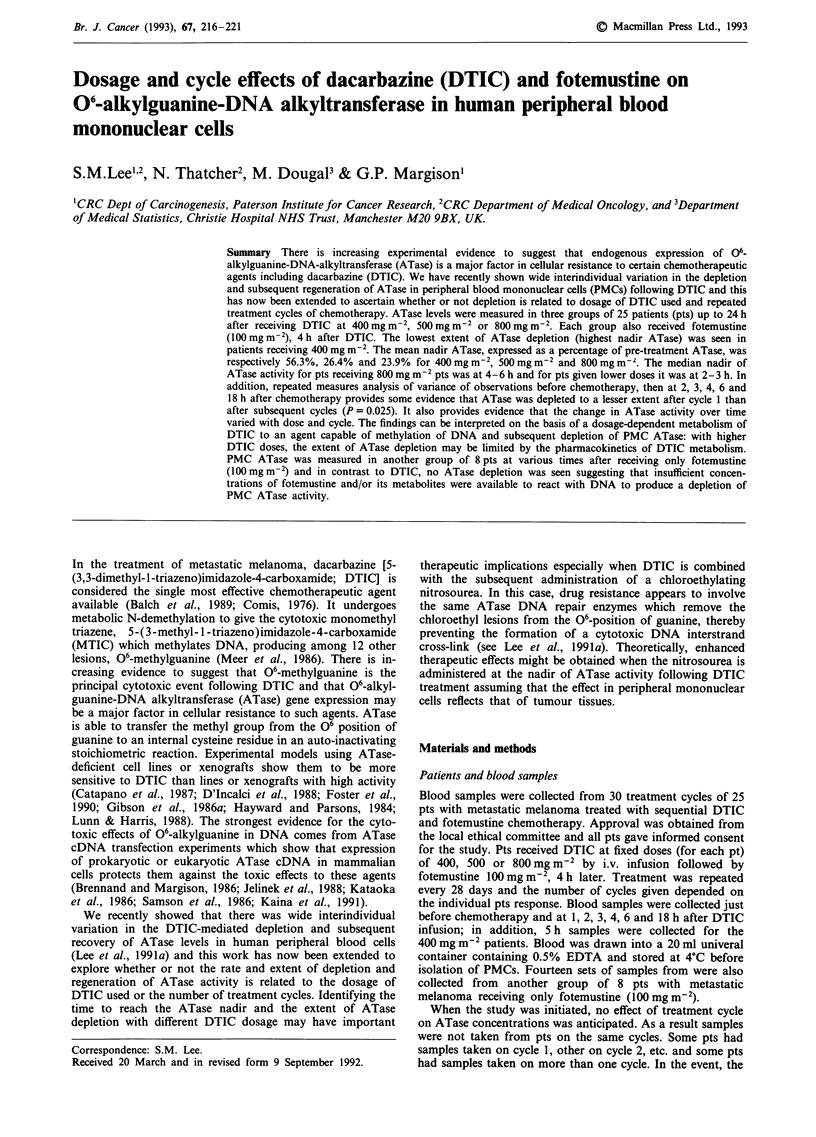

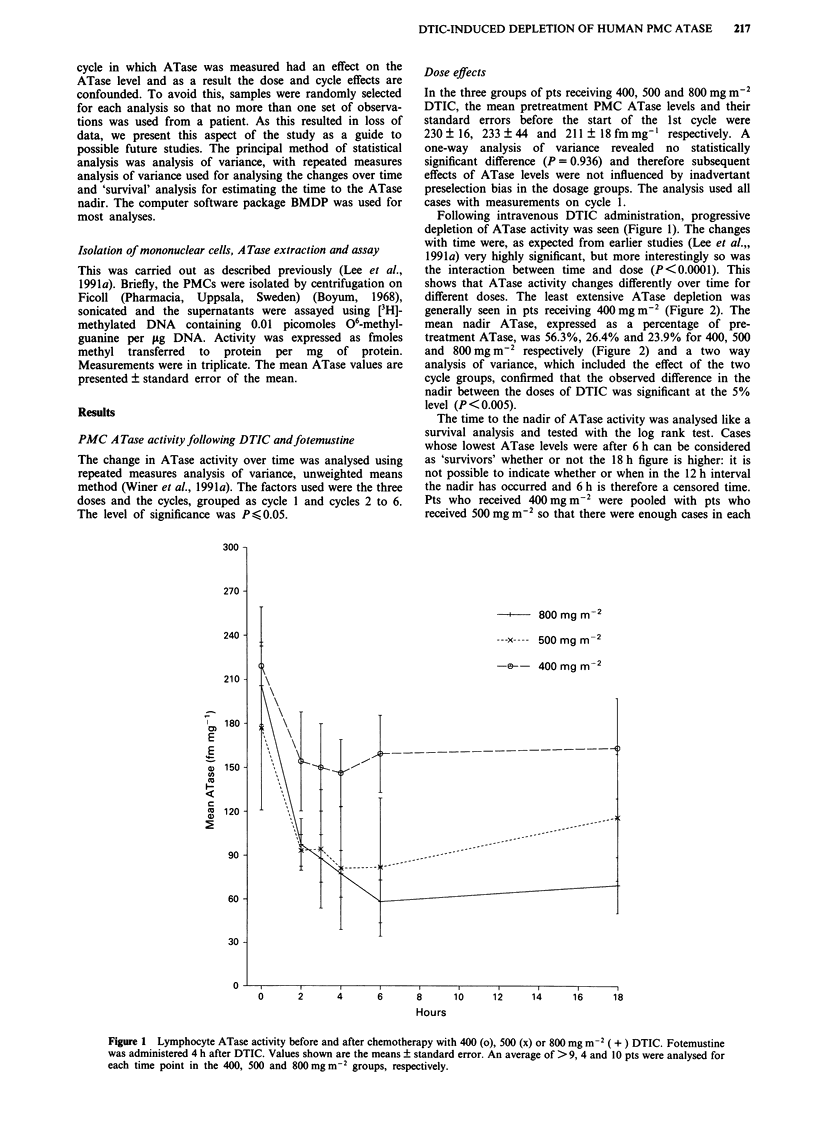

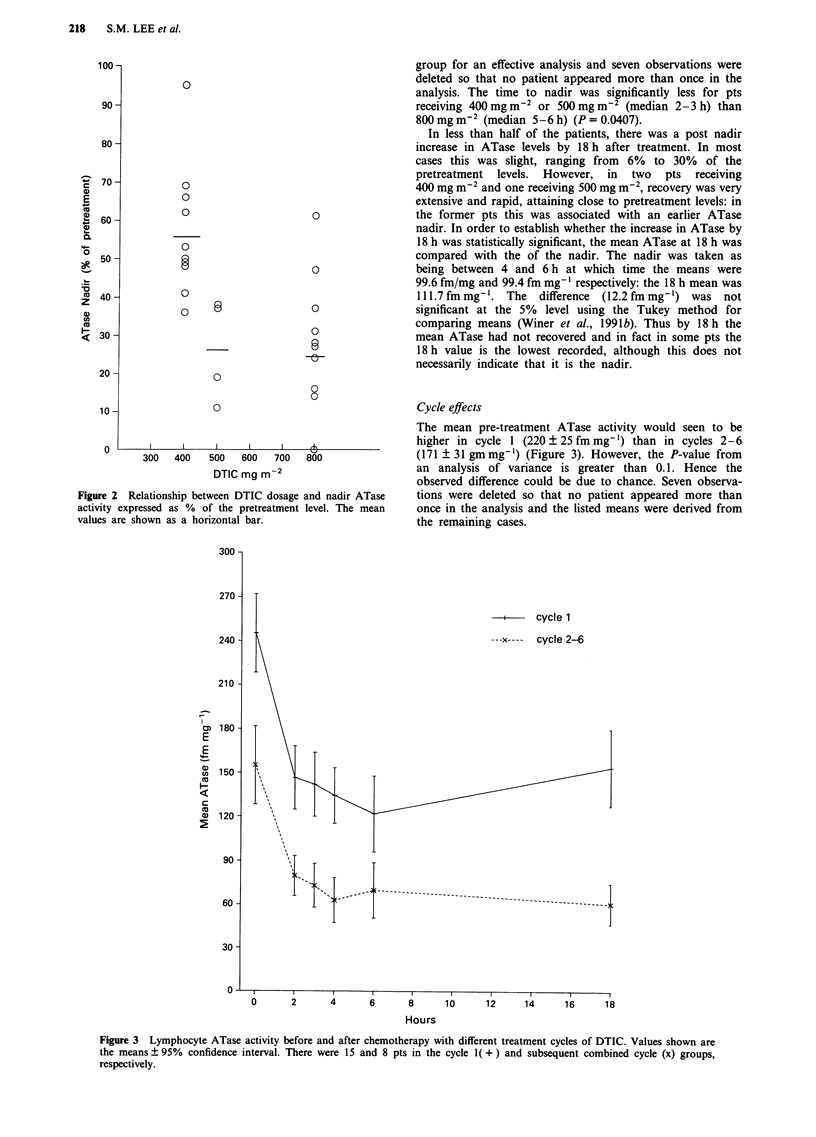

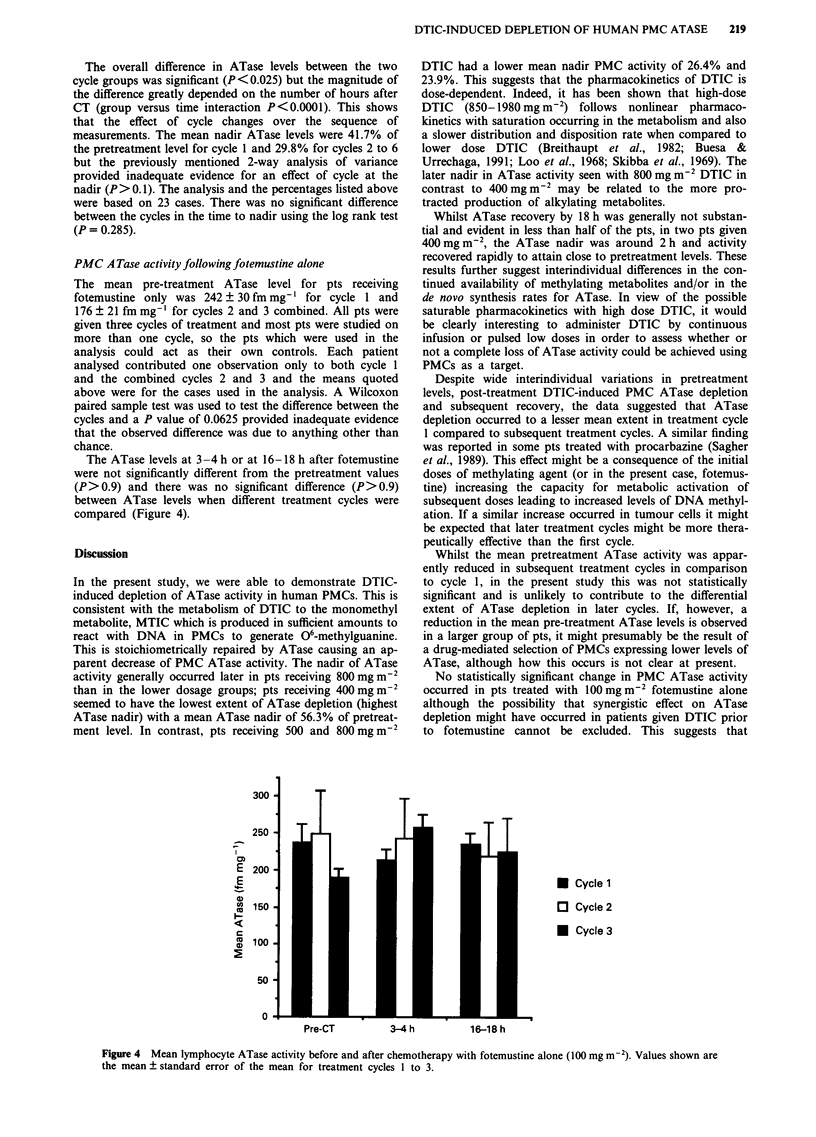

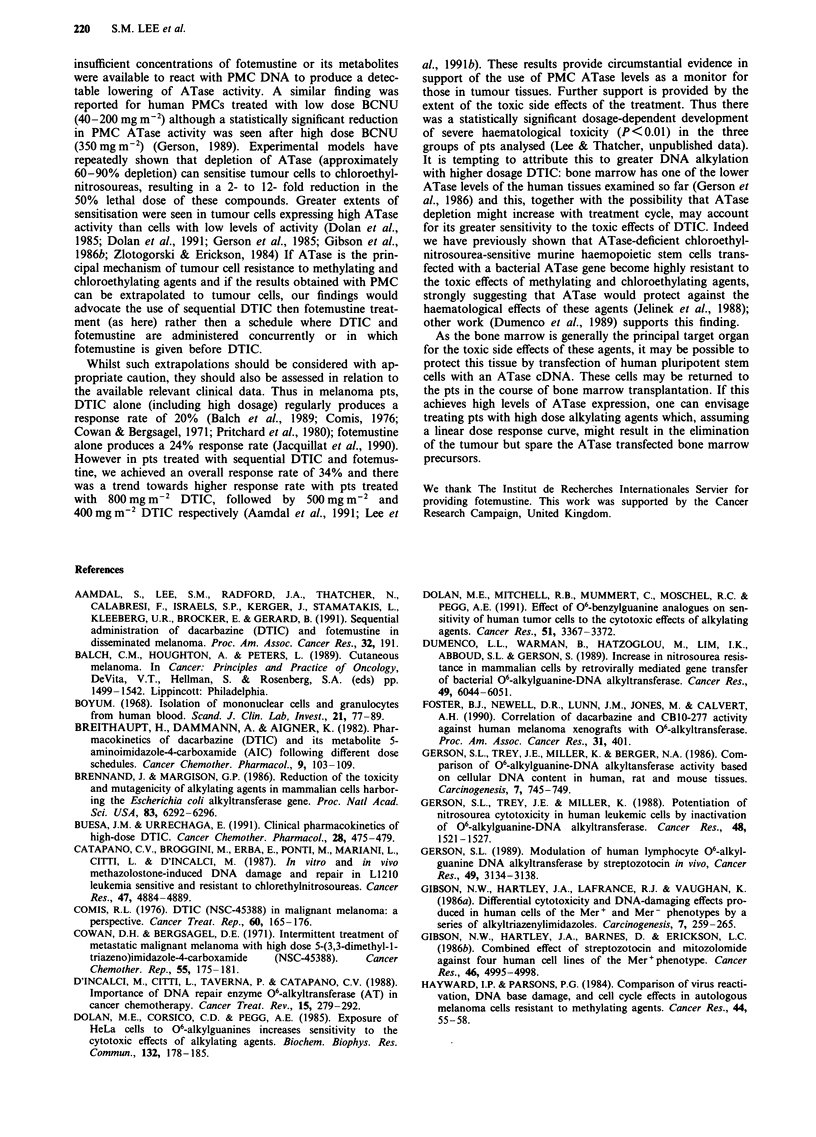

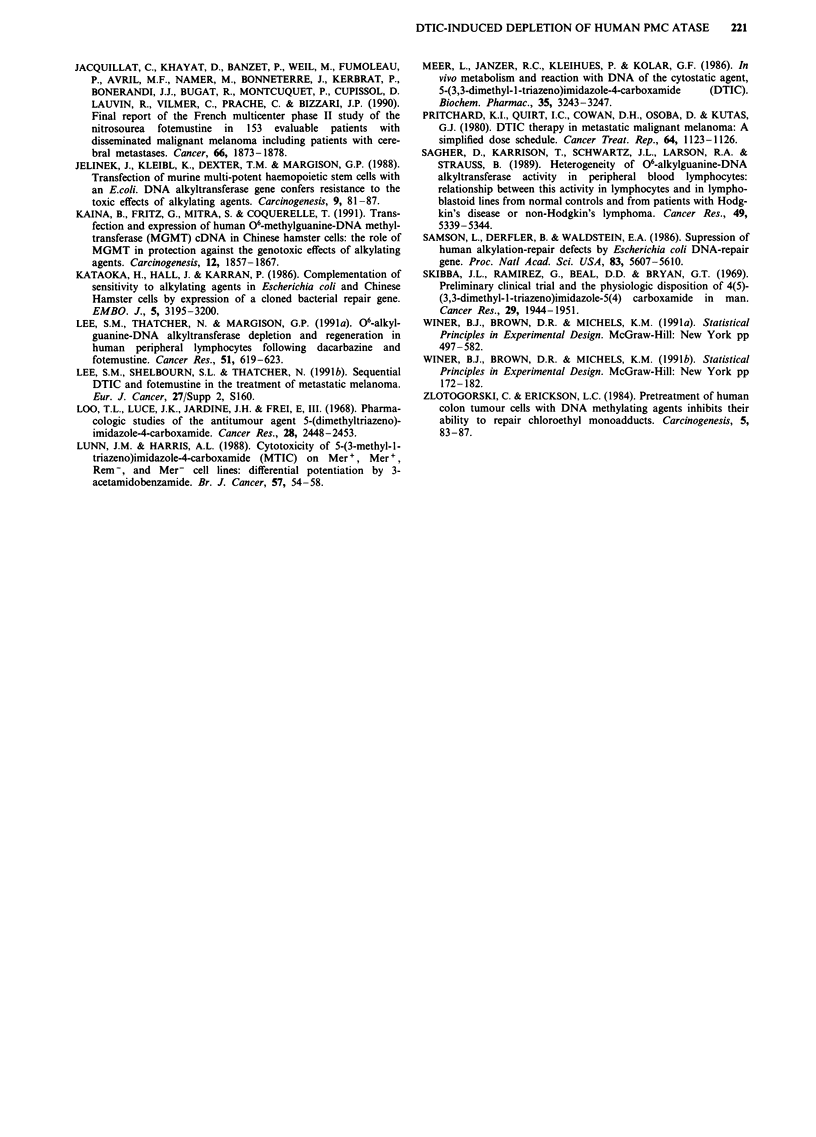


## References

[OCR_00593] Breithaupt H., Dammann A., Aigner K. (1982). Pharmacokinetics of dacarbazine (DTIC) and its metabolite 5-aminoimidazole-4-carboxamide (AIC) following different dose schedules.. Cancer Chemother Pharmacol.

[OCR_00599] Brennand J., Margison G. P. (1986). Reduction of the toxicity and mutagenicity of alkylating agents in mammalian cells harboring the Escherichia coli alkyltransferase gene.. Proc Natl Acad Sci U S A.

[OCR_00605] Buesa J. M., Urréchaga E. (1991). Clinical pharmacokinetics of high-dose DTIC.. Cancer Chemother Pharmacol.

[OCR_00589] Böyum A. (1968). Isolation of mononuclear cells and granulocytes from human blood. Isolation of monuclear cells by one centrifugation, and of granulocytes by combining centrifugation and sedimentation at 1 g.. Scand J Clin Lab Invest Suppl.

[OCR_00609] Catapano C. V., Broggini M., Erba E., Ponti M., Mariani L., Citti L., D'Incalci M. (1987). In vitro and in vivo methazolastone-induced DNA damage and repair in L-1210 leukemia sensitive and resistant to chloroethylnitrosoureas.. Cancer Res.

[OCR_00616] Comis R. L. (1976). DTIC (NSC-45388) in malignant melanoma: a perspective.. Cancer Treat Rep.

[OCR_00620] Cowan D. H., Bergsagel D. E. (1971). Intermittent treatment of metastatic malignant melanoma with high-dose 5-(3,3-dimethyl-1-triazeno)imidazole-4-carboxamide (NSC-45388).. Cancer Chemother Rep.

[OCR_00626] D'Incalci M., Citti L., Taverna P., Catapano C. V. (1988). Importance of the DNA repair enzyme O6-alkyl guanine alkyltransferase (AT) in cancer chemotherapy.. Cancer Treat Rev.

[OCR_00631] Dolan M. E., Corsico C. D., Pegg A. E. (1985). Exposure of HeLa cells to 0(6)-alkylguanines increases sensitivity to the cytotoxic effects of alkylating agents.. Biochem Biophys Res Commun.

[OCR_00637] Dolan M. E., Mitchell R. B., Mummert C., Moschel R. C., Pegg A. E. (1991). Effect of O6-benzylguanine analogues on sensitivity of human tumor cells to the cytotoxic effects of alkylating agents.. Cancer Res.

[OCR_00643] Dumenco L. L., Warman B., Hatzoglou M., Lim I. K., Abboud S. L., Gerson S. L. (1989). Increase in nitrosourea resistance in mammalian cells by retrovirally mediated gene transfer of bacterial O6-alkylguanine-DNA alkyltransferase.. Cancer Res.

[OCR_00668] Gerson S. L. (1989). Modulation of human lymphocyte O6-alkylguanine-DNA alkyltransferase by streptozotocin in vivo.. Cancer Res.

[OCR_00656] Gerson S. L., Trey J. E., Miller K., Berger N. A. (1986). Comparison of O6-alkylguanine-DNA alkyltransferase activity based on cellular DNA content in human, rat and mouse tissues.. Carcinogenesis.

[OCR_00662] Gerson S. L., Trey J. E., Miller K. (1988). Potentiation of nitrosourea cytotoxicity in human leukemic cells by inactivation of O6-alkylguanine-DNA alkyltransferase.. Cancer Res.

[OCR_00679] Gibson N. W., Hartley J. A., Barnes D., Erickson L. C. (1986). Combined effects of streptozotocin and mitozolomide against four human cell lines of the Mer+ phenotype.. Cancer Res.

[OCR_00673] Gibson N. W., Hartley J., La France R. J., Vaughan K. (1986). Differential cytotoxicity and DNA-damaging effects produced in human cells of the Mer+ and Mer- phenotypes by a series of alkyltriazenylimidazoles.. Carcinogenesis.

[OCR_00685] Hayward I. P., Parsons P. G. (1984). Comparison of virus reactivation, DNA base damage, and cell cycle effects in autologous human melanoma cells resistant to methylating agents.. Cancer Res.

[OCR_00697] Jacquillat C., Khayat D., Banzet P., Weil M., Fumoleau P., Avril M. F., Namer M., Bonneterre J., Kerbrat P., Bonerandi J. J. (1990). Final report of the French multicenter phase II study of the nitrosourea fotemustine in 153 evaluable patients with disseminated malignant melanoma including patients with cerebral metastases.. Cancer.

[OCR_00703] Jelinek J., Kleibl K., Dexter T. M., Margison G. P. (1988). Transfection of murine multi-potent haemopoietic stem cells with an E. coli DNA alkyltransferase gene confers resistance to the toxic effects of alkylating agents.. Carcinogenesis.

[OCR_00709] Kaina B., Fritz G., Mitra S., Coquerelle T. (1991). Transfection and expression of human O6-methylguanine-DNA methyltransferase (MGMT) cDNA in Chinese hamster cells: the role of MGMT in protection against the genotoxic effects of alkylating agents.. Carcinogenesis.

[OCR_00716] Kataoka H., Hall J., Karran P. (1986). Complementation of sensitivity to alkylating agents in Escherichia coli and Chinese hamster ovary cells by expression of a cloned bacterial DNA repair gene.. EMBO J.

[OCR_00722] Lee S. M., Thatcher N., Margison G. P. (1991). O6-alkylguanine-DNA alkyltransferase depletion and regeneration in human peripheral lymphocytes following dacarbazine and fotemustine.. Cancer Res.

[OCR_00733] Loo T. L., Luce J. K., Jardine J. H., Frei E. (1968). Pharmacologic studies of the antitumor agent 5-(dimethyltriazeno)imidazole-4-carboxamide.. Cancer Res.

[OCR_00738] Lunn J. M., Harris A. L. (1988). Cytotoxicity of 5-(3-methyl-1-triazeno)imidazole-4-carboxamide (MTIC) on Mer+, Mer+Rem- and Mer- cell lines: differential potentiation by 3-acetamidobenzamide.. Br J Cancer.

[OCR_00744] Meer L., Janzer R. C., Kleihues P., Kolar G. F. (1986). In vivo metabolism and reaction with DNA of the cytostatic agent, 5-(3,3-dimethyl-1-triazeno)imidazole-4-carboxamide (DTIC).. Biochem Pharmacol.

[OCR_00750] Pritchard K. I., Quirt I. C., Cowan D. H., Osoba D., Kutas G. J. (1980). DTIC therapy in metastatic malignant melanoma: a simplified dose schedule.. Cancer Treat Rep.

[OCR_00755] Sagher D., Karrison T., Schwartz J. L., Larson R. A., Strauss B. (1989). Heterogeneity of O6-alkylguanine-DNA alkyltransferase activity in peripheral blood lymphocytes: relationship between this activity in lymphocytes and in lymphoblastoid lines from normal controls and from patients with Hodgkin's disease or non-Hodgkin's lymphoma.. Cancer Res.

[OCR_00764] Samson L., Derfler B., Waldstein E. A. (1986). Suppression of human DNA alkylation-repair defects by Escherichia coli DNA-repair genes.. Proc Natl Acad Sci U S A.

[OCR_00769] Skibba J. L., Ramirez G., Beal D. D., Bryan G. T. (1969). Preliminary clinical trial and the physiologic disposition of 4(5)-(3,3-dimethyl-1-triazeno)imidazole-5(4)-carboxamide in man.. Cancer Res.

[OCR_00785] Zlotogorski C., Erickson L. C. (1984). Pretreatment of human colon tumor cells with DNA methylating agents inhibits their ability to repair chloroethyl monoadducts.. Carcinogenesis.

